# Fingerspelling as a pathway to deaf children’s reading: a scoping review

**DOI:** 10.1093/jdsade/enaf065

**Published:** 2025-10-31

**Authors:** Krister Schönström, Ingela Holmström

**Affiliations:** Department of Linguistics, Stockholm University, Stockholm, Sweden; Department of Linguistics, Stockholm University, Stockholm, Sweden

## Abstract

The role of fingerspelling or manual alphabet for reading among deaf and hard-of-hearing individuals has been of great interest in research. What can decades of research on fingerspelling and reading among deaf individuals tell us about how fingerspelling aids deaf children’s reading development? This scoping review was, therefore, undertaken to explore: (a) What is the relationship between fingerspelling and reading?, (b) In what ways do fingerspelling abilities promote reading skills?, and (c) What is known about using fingerspelling to teach reading? We identified 26 studies that were screened and summarized according to publication characteristics. The content and methods of these studies were briefly described. The results revealed a larger proportion of quantitative methods and ASL/English settings. A discussion and implications for future research are provided.

## Introduction

Previous literature in the area of reading and deaf education has reported that deaf students face different challenges when learning to read (e.g., Allen, 1994; Traxler, 2000). Different factors contributing to reading achievement in deaf students have been addressed in the scientific discussion; for example, access to spoken languages (e.g., Perfetti & Sandak, 2000), language difference (Mayer & Wells, 1996), sign language knowledge (Chamberlain & Mayberry, 2000), decoding strategies (Musselman, 2000), and more. However, it has also been reported that deaf students can be skilled readers/writers, although they typically acquire spoken languages via print (Hoffmeister & Caldwell-Harris, 2014; Schönström, 2010). Furthermore, researchers have repeatedly pointed out a positive relationship between sign language skills and reading skills (e.g., Chamberlain & Mayberry, 2008; Scott, 2022; Strong & Prinz, 1997). Importantly, this achievement can be complicated by the varied prerequisites and early language experiences of deaf students or learners (see, e.g., Howerton-Fox & Falk, 2019). Early access to a full and accessible language—whether spoken or signed—is crucial, as deaf children who experience early language deprivation are at a heightened risk of experiencing delays in linguistic, cognitive, and literacy skills (Gulati, 2018; Hall, 2017; see also Mayberry, 2007). Consequently, establishing a strong linguistic foundation early in life is essential for effective engagement with literacy-related skills.

There is a wide range of research showing that the use of sign language promotes deaf students’ learning of reading and writing (e.g., Hoffmeister & Caldwell-Harris, 2014; Scott, 2022; Scott & Hoffmeister, 2017, 2018). Among other things, research in the deaf education field has suggested that using sign language is an effective instructional practice for teaching deaf students to read and write, particularly when employing the manual alphabet or fingerspelling (e.g., Padden & Gunsauls, 2003; Scott et al., 2019; Walsh-Aziz et al., 2024).

Fingerspelling is a linguistic feature of sign languages (see further below). It functions as a manual code within the broader context of a sign language, serving as a tool to represent written words through hand configurations corresponding to specific letters. Importantly, children do not typically learn fingerspelling in isolation; instead, their development of fingerspelling skills is suggested to be intertwined with their overall language acquisition (e.g., Lee & Secora, 2022; Padden, 2006). A robust sign language foundation, encompassing vocabulary, syntax, and phonological awareness (PA) within the signed modality, is crucial for the meaningful integration of fingerspelling into literacy development.

Research suggests that children with strong sign language competence tend to utilize fingerspelling more effectively as a bridge to reading (e.g., Allen, 2015; Lederberg et al., 2019; Stone et al., 2015). Conversely, children experiencing language deprivation—common among deaf children who may not have had early access to a rich language environment—often face delays in both sign language development and literacy. This raises the possibility that associations observed between fingerspelling and reading in some studies may be moderated or even limited by the child’s language development status.

Furthermore, it is noteworthy that children often learn the movement patterns of fingerspelled words before mastering the individual letter signs (e.g., Padden, 1991). This sequential acquisition emphasizes the importance of holistic language exposure, as understanding the meaning and structure within language appears to underpin successful literacy outcomes.

Also, the educational backgrounds of deaf children deserve a comment. There are few sign-bilingual classrooms worldwide (where both sign language and spoken/written language are used), and these are rarely documented in research (Dammeyer & Marschark, 2016; Lange et al., 2013). Despite this, there are some valuable resources documented in *The Routledge Handbook of Sign Language Pedagogy* (Rosen, 2019); specifically, the chapters including strategies for teaching deaf students reading and writing, including sign language–print strategies, fingerspelling, technology/multimedia use, and Reading-Thinking-Signing (see Simms & Andrews, 2019). The pedagogical practices related to writing also apply here, such as content- or meaning-based instruction, interactional approaches, scaffolding strategies, chaining, and contrastive and translation methods (see Schönström & Holmström, 2019). In addition, the practice includes more specific pedagogical approaches such as Strategic and Interactive Writing Instruction (SIWI) (Dostal & Wolbers, 2014), and models for teaching literacy to deaf signing students (Kuntze et al., 2014). In this, fingerspelling is a recurring theme in studies about deaf students’ reading. A number of interventions have been conducted in deaf education, including developing educational materials or implementing fingerspelling as part of reading practices (e.g., Schick et al., 2018). However, it should be noted that deaf education varies greatly worldwide and is characterized by diverse methodologies and varying levels of instructional quality (see, e.g., Dammeyer, 2025; Moores, 2010; Moores & Miller, 2009). Therefore, interpreting outcomes across studies is challenging. The ambiguity surrounding the “visual method,” which may include established sign languages or constructed language variants, further complicates comparisons to oral-based approaches. Recognizing this variability is essential when analyzing studies, as some lack clear descriptions of instructional practices and students’ language backgrounds. This limitation requires careful consideration of the specific teaching methods used and students’ language proficiencies, whether in spoken or signed modes, to accurately interpret the findings. Many deaf children may not have received adequate education, which also impacts the results.

Our project, “Promoting the reading of deaf students in sign language-based classrooms (ReadSign)”, which is funded by the Swedish Institute for Educational Research (Skolforskningsinstitutet, dnr 2022-00022), examines an intervention in Swedish sign-bilingual preschool classes. ReadSign is a joint project in which researchers cooperate with teachers from sign-bilingual deaf schools throughout Sweden to explore the impact of bilingual fingerspelling-based educational material aimed at developing reading in Swedish. Within the project, we examine the relationship between sign language and early reading development in deaf children. We focus on fingerspelling and how it promotes metalinguistic knowledge and PA of language in deaf children as a pathway to improved reading skills. The working languages in the classes are Swedish Sign Language (*Svenskt teckenspråk*, STS) and written Swedish.

There are a few previous reviews covering reading in deaf children, specifically focusing on decoding (e.g., Musselman, 2000) and PA (Mayberry et al., 2011). Additionally, a valuable summary of fingerspelling research published in memoriam of Amy Hile’s work (Easterbrooks, 2017) provides an excellent, albeit brief, overview of early fingerspelling acquisition in deaf children. Phonological awareness—i.e., the ability to recognize and manipulate the internal structure of language, such as phonemes, syllables, and rhymes in spoken language, or handshape, movement, and location in sign languages—it is fundamental in both modalities. This cross-modal aspect of PA highlights its universal role in language development and literacy.^1^

To further explore the relationship between fingerspelling and learning to read, there is motivation to undertake a scoping review of literature reporting previous research on fingerspelling and reading development, particularly from a classroom perspective. Before delving into the scoping review, we will provide background information on fingerspelling as part of sign languages, core knowledge from studies on adults from a cognitive perspective, and studies investigating the effect of fingerspelling on groups other than the deaf.

### Fingerspelling as part of sign languages

Manual alphabets, or fingerspelling, is part of many sign languages and their lexicons. The formal properties of the manual alphabet differ between sign languages, with some sign languages using one-handed manual alphabets, such as American Sign Language (ASL) and STS. In contrast, others use two-handed manual alphabets, such as British Sign Language (BSL) and the older Norwegian Sign Language variant. Many manual alphabets in sign languages (e.g., ASL, Danish Sign Language, DSL, and Finnish Sign Language, FinSL) share similar formations of manual letters (“hand shapes”), which can be traced back in the French sign language family, for example (Power, 2022; Power et al., 2020), which is similar to the letters used in the international manual alphabet (see also Bergman & Engberg-Pedersen, 2010; Tapio, 2013). Historically, the origins of fingerspelling in sign languages seem to have been a product of intervention in early deaf education (Padden & Gunsauls, 2003; see also Geraci, 2015). For example, the founder of the first deaf school in Sweden, Pär Aron Borg, created the Swedish manual alphabet (and also implemented it in Portugal) to facilitate deaf students’ learning (Bergman & Engberg-Pedersen, 2010). Manual alphabets were used and implemented in several sign languages as time passed. However, their use and frequency seem to differ between sign languages and users. For example, ASL, together with STS and BSL, use fingerspelling frequently, while others, such as FinSL and Italian Sign Language, seem to use fingerspelling to a lesser extent (see, e.g., Brennan, 2001; Geraci, 2015; Padden & Gunsauls, 2003; cf. Börstell et al., 2016). According to the data in Padden and Gunsauls (2003), the use of fingerspelling in ASL varies between 12% and 35%, which is considerably high compared to the data presented in Börstell et al. (2016), in which the amount of fingerspelling varied between 3.0% and 6.4% for BSL, STS, Australian Sign Language (Auslan), and ASL. Obviously, more data (ideally larger corpus data) and more studies are needed to confirm the amount of fingerspelling used in sign languages cross-linguistically.

The functions of fingerspelling vary depending on the category and the signers’ sociolinguistic backgrounds. For ASL, Padden and Gunsauls (2003) found that nouns were more frequently fingerspelled than other categories, and that the use of fingerspelling varied depending on age, gender, work, education, and linguistic background. This has also been reported for Auslan in Schembri and Johnston (2007), which found that 10% of the lexical items in Auslan data were products of fingerspelling, and that the amount of fingerspelling varied depending on context and sociolinguistic factors (age or region).

The implementation of fingerspelling in sign lexicons can vary. For ASL, fingerspelling occurs in different proposed categories, such as initialized signs, abbreviation signs, loan signs, lexicalized fingerspelling, and neutral fingerspelling (Keane & Brentari, 2016; see also Battison, 1978). Similar categories can be found for STS (Bergman & Wikström, 1981). The handshapes of the manual alphabet letters can be either iconic (e.g., C for ASL and BSL) or non-iconic (e.g., F for ASL and STS) in how the handshapes resemble the form of the alphabet letters (Börstell, 2024; Lee & Secora, 2022).

The use of fingerspelling can be treated from other viewpoints, as well. For example, from a bilingual or multilingual perspective, signers can use fingerspelling to refer to words from different spoken languages as part of their communicative activities. Fingerspelling is also a frequent component in sign-bilingual teaching, often manifested as “chaining” (i.e., linking fingerspelling, signs, and written words) (Humphries & MacDougall, 1999; see also Bagga-Gupta, 2004; Morere & Roberts, 2012; Tapio, 2019). In recent years, the engagement of fingerspelling in multilingual contexts has been described within the translanguaging framework as one of the different components in the multilingual multimodal repertoire, such as in intramodal translanguaging; i.e., using the sign mode to spell spoken/written elements (Holmström & Schönström, 2018). From that perspective, fingerspelling is suggested to blur the lines between spoken, sign, and written languages, contributing to fluid translanguaging (Lee & Secora, 2022).

According to the research on acquiring ASL fingerspelling, deaf children in deaf families often begin to fingerspell early, as deaf parents often use fingerspelling as a natural part of their signing (Padden, 2006; Padden & Le Master, 1985). Also, fingerspelling is a natural part of bilingualism in families and schools (Blumenthal-Kelly, 1995; Erting et al., 2000; Maxwell, 1998; Padden, 1991, 2006; Padden & Le Master, 1985). It is suggested that deaf children seem to learn to fingerspell twice. First, the children’s perception of fingerspelling is uniquely related to ASL, just as other ASL signs occur in natural ASL conversations. Second, when the children come into contact with English and its orthography, they will start to relate the fingerspelling to written letter representations in English. The initial connection between fingerspelling and ASL differs for children and adult learners. While adult learners, such as second language learners of ASL, can connect letters with sign language fingerspelling from the very beginning, fingerspelled words are not obvious for deaf children in the beginning and require time to develop (Padden, 1991).

### Cognitive perspectives on fingerspelling and reading

Several studies have explored the relationship between fingerspelling and reading from a cognitive perspective, focusing primarily on deaf adults. Emmorey and Petrich (2012) examined how printed and fingerspelled words are interpreted concerning orthography and their effect on reading skills. Their study aimed to determine whether similar orthographic strategies were used for reading printed and fingerspelled words concerning segmentation and the potential for transfer effects. They discovered that while orthographic segmentation strategies differ between print and fingerspelling, indicating no transfer effect, there remains a significant correlation between fingerspelling and reading skills. This suggests that the connection is functional rather than sublexical.

Sehyr et al. (2017) delved into the differences in memory processing and linguistic recall between hearing and deaf individuals, with a focus on deaf ASL signers. They found that deaf signers recode printed and fingerspelled words into a phonological code, similar to hearing readers, although fingerspelling provides an alternative strategy by using hand gestures to represent words. This method may facilitate the phonological processing of printed material. Their findings highlight a strong connection between fingerspelling and English phonology, suggesting that teaching strategies that associate signs, fingerspelling, and print can improve vocabulary and reading skills in deaf children.

Further research by Sehyr and Emmorey (2022) examined how lexical quality—comprising phonology, orthography, and semantics—and ASL-related skills such as fingerspelling and comprehension contribute to reading comprehension in both deaf and hearing adults. They found that orthographic skills, rather than phonological reliance, were the strongest predictors of reading comprehension for deaf readers. Notably, the fingerspelling of real words—but not pseudowords—significantly predicted reading comprehension, reinforcing that real words form robust associations between their fingerspelled and written forms during vocabulary acquisition. The study suggests that deaf readers focus more on orthography and meaning rather than phonology.

In a recent neuroimaging study using Event-Related Potentials, Lee et al. (2025) explored the relationship between printed words, fingerspelling, and signs in skilled deaf readers. Their results showed that the semantic priming effect was larger and occurred more quickly for fingerspelling compared to signs. They propose that fingerspelling and printed words share orthographic coding, indicating that fingerspelling may play a crucial role in reading development for deaf children.

Stone et al. (2015) investigated reading fluency in deaf adults using various assessments, including reading fluency, ASL proficiency, fingerspelling skills, working memory, and cognitive functioning. Their findings revealed that fingerspelling skills were a significant predictor of reading fluency in deaf adults, surpassing other factors like ASL proficiency and age of acquisition. They noted that rapid and accurate decoding of fingerspelled words is critical for reading fluency. The researchers connected this to the Simple View of Reading model, suggesting that fingerspelling is linked to the decoding aspect of reading, whereas ASL proficiency relates to linguistic competence.

### Fingerspelling in other groups: hearing and disabled people

Over the years, primarily in the 1970s and 1980s, several studies have shown interest in the use of fingerspelling (and sometimes sign language) to support hearing children and youths with learning or reading disabilities (see, e.g., Blackburn et al., 1984; McKnight, 1979; Vernon et al., 1980, Walker, 1977; Wilson et al., 1985; for a compilation of earlier studies, see also Koehler & Lloyd, 1986). These studies address teaching challenges and explore alternative methods to improving learning outcomes. For example, McKnight (1979) emphasizes the importance of a strong phonetic approach for students with learning disabilities and explores a method where hand signs for letter sounds are introduced alongside the sounds themselves. This multisensory approach aims to improve the connection between sounds and visual symbols. McKnight found the advantages of such a method were reflected in students’ improved attention, teachers’ early detection of errors, and that the simultaneous use of sounds and signs facilitates repetition and reinforces learning.

Blackburn et al. (1984) also refer to research suggesting that multisensory approaches (incorporating tactile–kinesthetic input alongside auditory and visual input) can benefit some children with reading difficulties. They present a case study of two adolescent boys with severe reading difficulties. Using fingerspelling and sign language, both boys made significant progress in their reading skills over a 5-month period. However, several studies stress the need for further research to confirm the effectiveness of manual communication/alphabets and to understand which children benefit the most from this approach. In addition, several studies suggest that the use of fingerspelling primarily enhances student motivation and the enjoyment of reading.

Several studies conducted during the same decades primarily focused on hearing children with deaf parents (see, e.g., Griffith, 1985; Orlansky & Bonvillian, 1985; Prinz & Prinz, 1979, 1981; Wilbur & Jones, 1974), showing that they often acquire sign language before spoken language, developing both as separate systems rather than translating one to the other. Interestingly, as they were exposed to both sign and spoken languages during childhood, they were found to acquire vocabulary and grammatical structures faster than children who were only exposed to spoken language. These children often demonstrate impressive bilingual skills, including code-switching.

More recent research suggests that fingerspelling may also be used as a tool to help hearing children (without learning or reading disabilities or difficulties) decode English print.

For example, Daniels (2001) was interested in “how sign language can be used to improve hearing children’s English vocabulary, reading ability, spelling proficiency, self-esteem, and comfort with expressing emotions” (p. 3). Among other things, she mentions fingerspelling as a valuable precursor that lays a strong foundation for reading and spelling skills by leveraging the unique benefits of kinesthetic learning. Daniels highlights that fingerspelling provides a quicker and easier way for young children to learn to read than printing letters, reducing frustration and improving speed. For example, Daniels means that combining physical (fingerspelling) and cognitive (thinking about letters and sounds) activities strengthens memory and creates stronger associations between letters, sounds, and word meanings. And like earlier research, Daniels argues that fingerspelling engages multiple senses (visual, kinesthetic, auditory), reinforcing learning more effectively than visual-only methods like printing. In addition, Daniels suggests that, for hearing children, simultaneously speaking and fingerspelling reinforces the connection between sounds and letter forms.

### The current study

From the literature review above, it can be concluded that the use of a manual alphabet is part of many sign languages and is frequently used by many sign language users. However, the function and frequency of fingerspelling can vary depending on a wide set of factors.

Cognitive studies on deaf adults have shown a positive relationship between fingerspelling and reading skills, which is connected to decoding strategies, emphasizing the importance of orthographic and sign language-based strategies in literacy instruction for deaf learners. In studies on hearing and disabled people, fingerspelling is suggested to have a kinesthetic effect and engages multi-sensory sources that facilitate learning. The research shows that deaf readers can achieve strong reading skills through alternative pathways, offering valuable insights for improving literacy education for both deaf children and adults by focusing on their strengths and adapting teaching methodologies accordingly.

However, there is a need to better understand how fingerspelling is related to reading skills in deaf and hard-of-hearing (DHH) children, especially in classroom contexts, and what results have been reported. Therefore, the objective of this scoping review is to account for the existing knowledge about the role of fingerspelling in reading development in DHH children based on the existing body of research conducted over the past few decades. The primary research question is, broadly: What does research tell us about how fingerspelling is linked to DHH children’s reading development? We formulate this question relatively broadly in order to screen existing literature about fingerspelling and reading. Focusing on the scope of the study, we are specifically interested in addressing the following questions: (a) What is the relationship between fingerspelling and reading?, (b) In what ways does fingerspelling promote reading skills?, and (c) What is known about the use of fingerspelling in teaching reading?

## Method

This scoping review of the literature about fingerspelling and reading in DHH children follows the JDSDE protocol for scoping review papers. In the interest of rigor in our review, we followed the guidelines and checklists in the PRISMA statement (http://www.prisma-statement.org/scoping) and the meta-analysis by Tricco et al. (2018). We used the Preferred Reporting Items for Systematic Reviews and Meta-Analyses extension for Scoping Reviews (PRISMA-ScR) Checklist as a template for our work.

### Systematic search strategy

Prior to our search, we consulted a university librarian for recommendations and suggestions on the technical merit of the search strategy. In February 2024, we searched the following databases: Web of Science, Scopus, Education Resources Information Center, Project Muse, PsycInfo, and Linguistics and Language Behavior Abstracts. Consequently, this review does not include anything published after the end of February 2024.

We searched the following concepts: sign language, fingerspelling, manual alphabet, hand alphabet, child, adolescent, teenager, deaf, hard-of-hearing, reading, and literacy. The exact search strings and combinations used can be found in Table 1.

**Table 1:** Search words and strings used in the search.

deaf* OR “hearing impaired” OR “hard-of-hearing” OR “hard of hearing”					
AND					
child* OR youth* OR teenager* OR adolescent*					
AND					
reading OR literacy					
AND					
“sign language” OR “manual alphabet” OR fingerspelling OR “hand alphabet”					

In addition, due to our origins and language knowledge, we searched for publications in Swedish/Norwegian/Danish in the specific research databases for Swedish scientific publications (SwePub), Norwegian scientific publications (CRISTIN), and the Danish research portal, using equivalent search strings in Swedish, Norwegian, and Danish, respectively. Before this search, we consulted with our Scandinavian colleagues regarding appropriate databases in their respective countries.

### Inclusion and exclusion criteria

Following our research questions, we focused on: (a) studies that concentrated on and reported on the relationship between fingerspelling and reading, and (b) studies that described working with fingerspelling to facilitate reading in DHH children. Therefore, we excluded: (a) studies where fingerspelling was a variable but not directly related to reading, (b) studies about reading not directly related to fingerspelling, (c) studies that included non-DHH or adult participants, and (d) reviews or overviews of educational models including fingerspelling.

We did not limit the search to certain years. In fact, very few publications related to sign language and fingerspelling in general existed before the 1990s. We only included publications written in English, Swedish, Norwegian, and Danish. As our review focuses only on DHH children, we summarized other studies and results regarding hearing children and deaf adults in the background section above.

## Results

**Figure 1:**
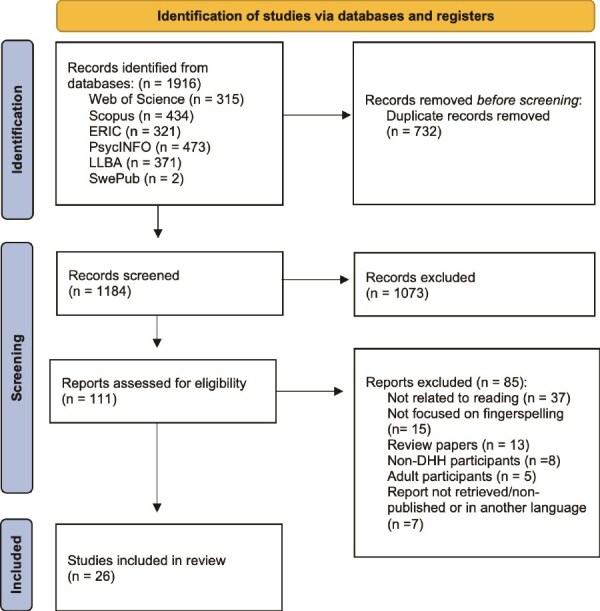
PRISMA flow diagram 2020 for search and selection process.

The search and selection process is presented as a flow diagram in Figure 1 according to the PRISMA flow diagram 2020 (Page et al., 2021). The first search resulted in 1,916 records. This included searches in the three Scandinavian databases that resulted in two records from the Swedish SwePub but none from the Norwegian and Danish databases. After deleting duplicate results, there were 1,184 records left. The records, including titles and abstracts, were imported to a database software, Zotero 6.0.37. After that, we narrowed our search in this database for “fingerspelling,” “manual alphabet,” and “hand alphabet.” In this search, there were no hits on “hand alphabet,” but 92 hits on “fingerspelling”, and 19 on “manual alphabet.” In total, we found 111 records that contain studies focusing on fingerspelling or on the manual alphabet.

Two reviewers (the authors) then screened all 111 records, and 26 publications which fit our research questions as described above were included in the analysis. In addition, we removed conference papers, presentations, and book chapters with the same content as a published journal article (albeit with another title), as we prioritized peer-reviewed journal articles. We included PhD dissertations but not MA theses.

We know that more publications in other countries and languages may exist. Still, due to our available language knowledge, our accounts are, as mentioned above, limited to publications in English and Scandinavian languages only.

### Data charting process

A data form was created in Microsoft Excel to organize the information obtained from our reading of the included publications. Each author read half of the publications (of a total of 26) and filled out information points in the Excel document, which included publication characteristics (author(s), title, year, journal) and study characteristics (demographic information, method, sample, study design, and core results). The results from the data charting process are presented in the next section, “publication characteristics.”

### Publication characteristics

We use a data charting process and a table listing the included publications in the review, see Table 2. Regarding quantitative versus qualitative methods, if a paper is mainly quantitative—i.e., its main results are based on quantitative data—we labeled it “quantitative”, and we followed the same pattern for papers based on qualitative data. If the main results are based on both quantitative and qualitative methods, or if the method is hybrid—i.e., combining experimental design with case study design—we categorized them as mixed methods.

**Table 2:** Publication characteristics of the studies included in the review.

*Author*	*Journal/Publication type*	*Method*	*N Sample*	*Classroom study*	*Participants*	*Age*	*Country*
Allen (2015)	Sign Language Studies	Quantitative	N = 237	No	Deaf	3–5 years	USA
Alvarado et al. (2007).	American Annals of the Deaf	Quantitative	N = 28 (and 15 controls).	No	Deaf (and hearing control group)	7–16 years	Chile
Bailes (1999).	Dissertation	Qualitative	N = 9	Partly	4 primary-grade teachers (3 deaf); 2 team leaders and 3 parents	n/a	USA
Crume et al. (2021)	Journal of Deaf Studies and Deaf Education	Quantitative	N = 32	No	Deaf	8;6–12;10	USA
Crume (2013).	Dissertation	Qualitative	N = 10	Partly	Teachers of the deaf (9) and an ASL specialist	n/a	USA
Erting and Pfau (1997).	Gallaudet University, Laurent Clerc National Deaf Education Center	Qualitative	N = 2	Partly	Two teachers of deaf children	n/a	USA
Hanson et al. (1984).	Journal of Experimental Child Psychology	Quantitative	N = 16	No	Deaf	6.25–11.0 years.	USA
Haptonstall-Nykaza and Schick (2007).	Journal of Deaf Studies and Deaf Education	Quantitative	N = 21	No	Deaf	4–14 years	USA
Harris and Beech (1998).	Journal of Deaf Studies and Deaf Education	Quantitative	N = 80	No	Deaf (24) and hearing (56)	Deaf: 4;02–6;02 years, Hearing: 4;11–5;09	England
Hile (2010).	Dissertation	Quantitative	N = 55	No	Deaf and Hard-of-Hearing	5–6 years and 8–9 years	USA
Hirsh-Pasek (1986).	Reading Teacher	Quantitative	N = 25	Partly	Deaf	5–16 years	USA
Hirsh-Pasek (1987).	Reading Research Quarterly	Quantitative	N = 26	No	Deaf	5–16 years (divided into two groups: 5–11 and 13–16)	USA
Lederberg et al. (2019).	Journal of Deaf Studies and Deaf Education	Quantitative	N = 336	No	Deaf	Pre-school up to secondary grade	USA
Lissi et al. (2009).	L1 Educational Studies in Language and Literature	Qualitative	Three teachers, eight video recordings of classroom activities	Yes	Deaf students, hearing teachers	Preschool–2nd grade students	Chile
MacGlaughlin (2018)	Dissertation	Qualitative	N = 3	No	Deaf	5–6	USA
Miller et al. (2021).	Journal of Deaf Studies and Deaf Education	Mixed methods	N = 4	Yes	Deaf	4.5–6.0 yrs	Israel
Ormel et al. (2022).	Languages	Quantitative	N = 64	Partly	Deaf (and hearing control group)	8–10 years	The Netherlands
Padden and Ramsey (1998).	Topics in Language Disorders	Mixed methods	Study 1: N = 31 Study 2: N = 7 Study 3: N = 2	Yes	Deaf and deaf/hearing teachers	Mean age 8.4 and 11.9	USA
Padden and Ramsey (2000).	Language acquisition by eye	Mixed methods	N = 31 and N = 7	Yes	Deaf and deaf/hearing teachers	4–5^th^ graders and 7–8^th^ graders	USA
Puente et al. (2006).	American Annals of the Deaf	Quantitative	N = 26	No	Deaf	7–10 and 12–15	Chile
Roos (2013).	Deafness and Education International	Qualitative	N = 6	Yes	Deaf	3:1–6:9 years	Sweden
Roos (2014).	Deafness & Education International	Qualitative	N = 6	Yes	Deaf	3:1–6:9 years	Sweden
Roos (2004).	Dissertation	Qualitative	N = 6	Yes	Deaf	3:1–6:9 years	Sweden
Scott et al. (2019).	American Annals of the Deaf	Mixed Methods	N = 6	Yes	CI/HA	Two groups: 6–7 years and 8–10 years	USA
van Staden and le Roux (2010).	Journal of Developmental and Physical Disabilities	Quantitative	Treatment group: N = 32 Comparison group: N = 32	Yes	Deaf	Mean age 117–119 months	South Africa
Wang and Andrews (2021).	American Annals of the Deaf	Qualitative	N = 6 and N = 5	Yes	Deaf	Two groups: 1^st^ graders and 2^nd^ graders	China

**Figure 2:**
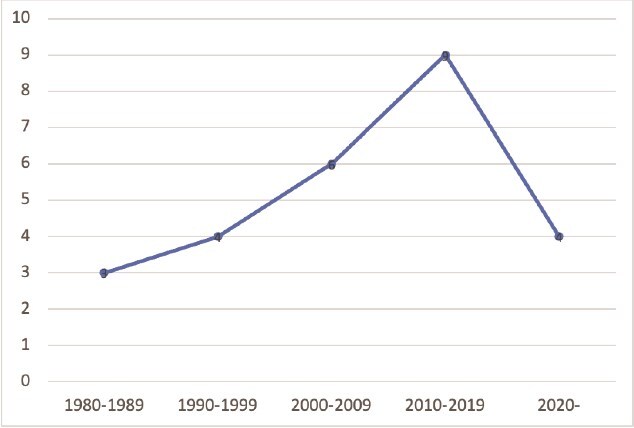
Number of publications per decade.

Figure 2 shows the number of fingerspelling research publications per decade from the 1980s onwards. Notably, half of the publications are from 2010 onwards, despite the fact that the 2020s is not yet a completed decade.

**Figure 3:**
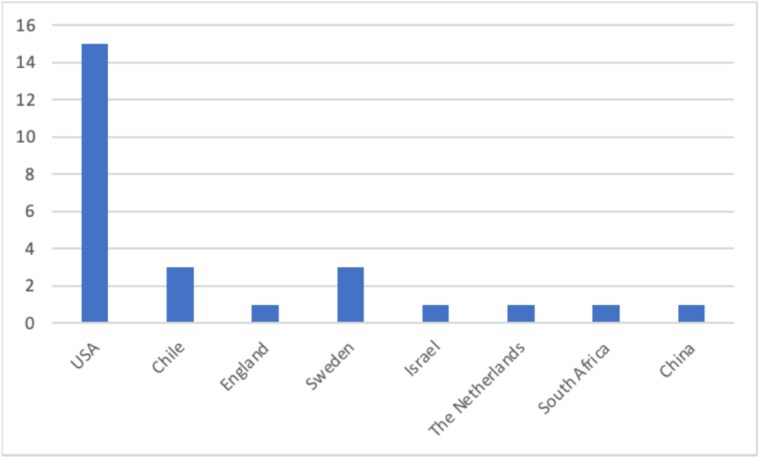
Number of publications according to country.

Figure 3 shows the number of publications published in each country. In total, eight countries are represented. Most publications are related to ASL and English, next to Chilean Sign Language (CHSL) and Spanish, as well as STS and Swedish, while other countries had one publication each.

Most publications are from peer-reviewed journals, except for five dissertations and one report.

### Methodological approaches in the studies

**Figure 4:**
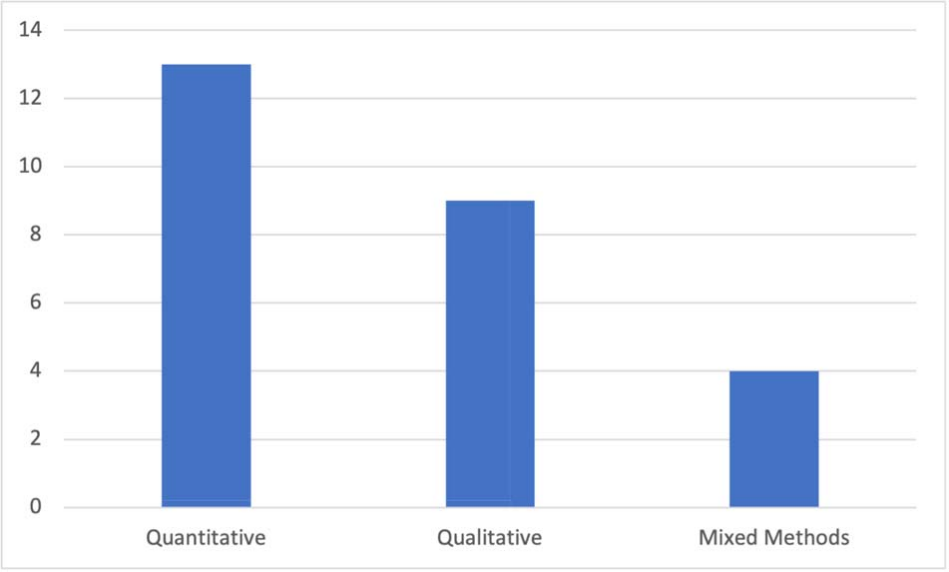
Methodological approach in the studies.

Regarding the methodology used in the reviewed studies, most results are based on quantitative and qualitative methods. Some studies used a mixed methods approach (see Figure 4). A total of 10 studies reported data from classrooms, five used data partly pulled from classrooms, and 11 studies were conducted outside classrooms. The vast majority of studies used data pulled directly from children. In contrast, some studies, specifically interview studies, used data from teachers of the deaf or parents to obtain information about fingerspelling and reading in children. Several studies combine data from children together with data from teachers/parents or data from control groups.

The study participants ranged in age from 3 to 16, and the number of participants ranged from 2 to 336. In the coming sections, we will account for the studies according to which method was used (quantitative, qualitative, or mixed).

### Quantitative studies

In total, 13 publications used a quantitative approach: Allen (2015); Alvarado et al. (2007); Crume et al. (2021); Hanson et al. (1984); Haptonstall-Nykaza and Schick (2007); Harris and Beech (1998); Hile (2010); Hirsh-Pasek (1986, 1987); Lederberg et al. (2019); Ormel et al. (2022); Puente et al. (2006); and van Staden and le Roux (2010). We will summarize each of the studies below.

#### Allen (2015)

Allen (2015) explored the connection between fingerspelling and early alphabetic knowledge in young deaf children; particularly how fingerspelling and ASL proficiency affect letter recognition and writing. The study used direct assessments, family surveys, and teacher questionnaires. A 13-item scale was created for teachers and parents to evaluate children’s fingerspelling skills, with tasks ranging from fingerspelling names to more complex integration of fingerspelled words in sentences. Teachers rated children’s abilities on a four-point scale. The analysis focused on teacher ratings as indicators of literacy progress. Results showed improvements in fingerspelling between ages three and five, with simpler skills developing faster. Gains in fingerspelling, especially name fingerspelling, were significant between ages four and five. The study confirmed that age and fingerspelling enhance letter knowledge, with ASL playing an important independent role in early language exposure for reading. Children from signing families developed letter knowledge similarly, while deaf children from hearing families showed varied results, indicating the need for more research to identify influencing factors.

#### Alvarado et al. (2007)

Alvarado et al. (2007) investigated whether novice (n = 13) and skilled deaf signers (n = 15), along with hearing controls (n = 15), employ different working memory coding strategies and how stimulus similarity affects memory recall. The study also examined the relationship between these coding strategies and reading skills among deaf Chilean signers. Participants were grouped based on their CHSL test performance. Experiment 1 involved recalling sequences of Spanish alphabet letters, while Experiment 2 explored coding strategies, including speechreading, dactylic handshapes (fingerspelling), and printed graphemes, in relation to grapheme recognition in signed words and reading skills. This experiment found a strong link between fingerspelling development and orthographic development. Results indicated that proficiency in sign language is the most significant predictor of reading level, supporting the notion that sign language proficiency enhances advanced literacy skills in a second language. Also, the study suggests that fingerspelling is a crucial element in coding words for deaf students, providing an alternative to phonological contrasts of oral language. It enables a connection between graphemes and handshapes.

#### Crume et al. (2021)

The study by Crume et al. (2021) involved 32 deaf students in grades three to six who completed nine tasks assessing their proficiency in ASL, English-influenced signing, and reading comprehension. One task was the Fingerspelling Ability and Phonological Awareness Test, which included five subtests designed for fingerspelling: (a) imitation of 12 fingerspelled words, (b) alliteration identification with pictures, (c) rhyming pairs of pictures, (d) elision by removing a letter from a fingerspelled word, and (e) blending two fingerspelled parts into a complete word. The researchers found strong correlations between fingerspelling phonological processing and reading comprehension (r = .81) and word reading (r = .88), indicating that fingerspelling aids in developing sublexical skills essential for reading. They concluded that fingerspelling helps bridge ASL and English, providing representations of English words lacking sign equivalents and supporting deaf children in learning to read.

#### Hanson et al. (1984)

Hanson et al. (1984) examined how 16 prelingually and profoundly deaf children (median age 8.75 years) remember printed letters, with a specific focus on the role of fingerspelling as a key linguistic code. The study examined whether children utilized fingerspelling alongside speech sounds to aid their short-term memory. The children were tested with letter groups that were dactylically similar (based on fingerspelling handshapes), phonetically similar, or visually similar, with a control group of unrelated letters. Results showed that successful readers employed both dactylic (fingerspelling) and phonetic codes for letter recall, demonstrating a linguistic strategy similar to that used by hearing children. Conversely, poor readers relied more on visual memory strategies instead of utilizing fingerspelling or phonetic coding. The study concluded that explicitly teaching deaf children to use fingerspelling as a memory strategy, in conjunction with any residual phonetic awareness, could enhance their reading skills and comprehension by providing a robust linguistic foundation.

#### Haptonstall-Nykaza and Schick (2007)

Haptonstall-Nykaza and Schick (2007) investigated how “lexicalized fingerspelling,” which incorporates sign-like movement patterns, can aid deaf children in learning new English vocabulary. The study involved 21 profoundly deaf children, aged 4–14, at a bilingual ASL-English school, who learned new words using two methods: (1) the Sign Condition, using ASL signs; and (2) the Fingerspelling Condition, employing lexicalized fingerspelling. Results indicated that children performed better in the Fingerspelling Condition, showing higher scores in recognizing, writing, and fingerspelling new words. Findings suggest that lexicalized fingerspelling acts as a “visual phonological bridge,” enhancing vocabulary learning and reading skills for deaf students.

#### Harris and Beech (1998)

Harris and Beech (1998) compared reading progress over two years between 24 deaf and 56 hearing children with similar IQs, focusing on skills affecting reading development. Deaf children received either oral-only instruction or Sign English, allowing researchers to observe the natural impact of signing and fingerspelling. Seven assessments were used, including PA, fingerspelling, and language comprehension. Generally, deaf children showed slower reading progress, especially in word recognition and implicit PA. Reading progress correlated with oral skills and language comprehension, particularly linked to signing and fingerspelling. The study found no direct link between fingerspelling and reading progress, indicating a need for fluent fingerspelling and further research.

#### Hile (2010)

Hile (2010) studied how deaf children learn familiar and novel fingerspelled words and how this skill relates to their reading and vocabulary. Fifty-five deaf children aged 5–6 and 8–9, from hearing and deaf families, completed a task involving fingerspelling common and nonsense words, followed by tests assessing their imitation, matching, production, recognition, and writing abilities. Results showed older children and those from deaf families outperformed others. Hile found strong links between fingerspelling skills and reading and vocabulary, especially among those with longer periods of ASL immersion. The study suggests that structured exposure to fingerspelling enhances language and literacy development in deaf children.

#### Hirsh-Pasek (1986 *,*  1987)

Hirsh-Pasek (1986, 1987) explored how native ASL students aged 5–16 connect printed words to language by using fingerspelling to decode print into English phonemes. For effective learning, students must recognize fingerspelled words, break them into handshapes, and map those handshapes to letters. The study included four experiments assessing whether focusing on fingerspelling supports early reading skills. The first three tasks tested students’ abilities to sort and manipulate handshapes, while the fourth involved matching printed and fingerspelled words to pictures. Results showed a strong link between metalinguistic skills and reading success, highlighting that fingerspelling can bridge gaps for deaf readers and support vocabulary development, which predicts reading achievement.

#### Lederberg et al. (2019)

Lederberg et al. (2019) studied how deaf and hard-of-hearing children learn to read, focusing on spoken-only, sign-only, and bimodal learners. They tested relationships between reading skills, language, spoken PA, and fingerspelling abilities in children from kindergarten to second grade. The researchers utilized a test battery that included speech perception, language assessments, and novel fingerspelling tasks, such as imitating fingerspelled words and blending handshapes. Results indicated that while all groups rely on language and word segmentation for reading, their modalities differ. Spoken-only and bimodal learners benefit from spoken PA, whereas sign-only learners use fingerspelling as a vital tool for connecting print to language, emphasizing the need for tailored reading instruction.

#### Ormel et al. (2022)

Ormel et al. (2022) conducted a 2-year longitudinal study on the effects of various skills on reading. They tested vocabulary, PA, fingerspelling, and short-term memory as predictors of word and reading fluency in 64 deaf children aged 8 and 10 from a sign bilingual education program in the Netherlands, along with 40 hearing age-matched controls. A developed fingerspelling recognition test assessed fingerspelling ability. The predictors were evaluated initially, while word and reading fluency were assessed after one and two years. Stepwise regression analysis revealed that age, speech-based vocabulary, and fingerspelling were the strongest predictors of reading fluency. Fingerspelling’s predictive power relates to its role in mapping handshapes to letters, facilitating orthographic skills like segmentation and decoding. Notably, fingerspelling correlated with sign vocabulary and PA, suggesting it serves as a bridge between signs and written words.

#### Puente et al. (2006)

Puente et al. (2006) examine the effectiveness of fingerspelling for reading and writing development in two Chilean Deaf groups, children (ages 7–10, n = 13) and adolescents (ages 12–15, n = 13) while controlling for reading skills. The research explored the association between fingerspelling and word identification, reading, spelling, and orthographic skills. Three experiments were conducted: (1) identifying CHSL signs and fingerspelled words, (2) matching fingerspelled words with logos, and (3) decoding fingerspelling to writing. Results showed that adolescents outperformed children in identification and matching tasks. Task performance correlated with reading skills; skilled readers did better overall. The authors concluded that fingerspelling and sign language skills facilitate reading and writing development, mediating between the alphabet and reading comprehension by enhancing knowledge of word structure.

#### van Staden and le Roux (2010)

This South African study by van Staden and le Roux (2010) tests the hypothesis that strong sign language skills and visual coding strategies can provide deaf children with a phonological or orthographic link to written language, enhancing their writing abilities. The study aimed to: (1) investigate whether fingerspelling supports English word learning, and (2) assess the efficacy of fingerspelling with visual imaging techniques. A quasi-experimental pre-test/post-test design involved a treatment group (n = 32, M(age) =119.19 months) and a comparison group (n = 32, M(age) =117 months) of deaf signing children with spelling difficulties in grades 1–3. After a year of fingerspelling coding and image-word mapping training, the treatment group outperformed the comparison group in spelling on post-tests, regardless of grade. The authors suggest combining visual coding strategies, including fingerspelling, facilitates learning written language by creating a phonological–orthographic link between sign language and written language.

### Qualitative studies

In the review, we identified nine articles as qualitative studies: Bailes (1999); Crume (2013); Erting and Pfau (1997); Lissi et al. (2009); MacGlaughlin (2018); Roos (2004, 2013, 2014); Wang and Andrews (2021). We will account for these studies below.

#### Bailes (1999)

Bailes’ (1999) dissertation investigates how teachers at a bilingual school for deaf children utilize ASL to teach English literacy, focusing on ASL as the first language and metalinguistic awareness. Through semi-structured interviews, classroom observations, and document reviews with four teachers, two team leaders, and three parents, the study shows that fingerspelling bridges ASL and English. Teachers used techniques like “chaining” (fingerspelling, signing, writing) and “sandwiching” (fingerspelling, signing, repeating fingerspelling) to reinforce English vocabulary and grammar, especially in areas where ASL lacks direct equivalents. Fingerspelling enhanced students’ recall and comprehension, making it a crucial instructional tool for connecting English language learning with ASL.

#### Crume (2013)

Crume's (2013) dissertation explores how teachers in a bilingual preschool program for deaf children use ASL to facilitate learning and connect it to English. The study focuses on teaching PA in ASL, with an emphasis on handshapes and signs aiding English learning. Crume interviewed nine teachers and one ASL specialist, supplementing the interviews with surveys. Teachers employed strategies like fingerspelling to bridge ASL and English, believing that understanding ASL’s structure enhances language comprehension. Younger students learned ASL basics, while older students connected ASL to English using “chaining” and “lexicalized fingerspelling,” where common fingerspelled words become more sign-like. Despite preschoolers’ challenges with lexicalized fingerspelling, older children found it to be effective. Ultimately, early exposure to ASL phonology, fingerspelling, and chaining improved language connections, making learning more visual and engaging.

#### Erting and Pfau (1997)

In this article, Erting and Pfau (1997) discuss—or engage in “theoretically informed speculation” (i.e., using theory and research to reflect on their own teaching experiences)—how to support emerging literacy in deaf preschoolers through bilingualism in ASL and English, drawing from both existing research and their teaching practices. The focus is on three key areas: developing metalinguistic awareness (understanding both languages), shared storybook experiences, and writing development. Erting and Pfau emphasize that building connections between ASL and English takes time, and involves strategies such as fingerspelling to link handshapes with letters and words. Fingerspelling is primarily highlighted for reading, not writing. Social interaction and environmental print (written words and symbols children encounter in their everyday surroundings) are also important for literacy development. One example provided by the authors illustrates how a teacher used fingerspelling to clarify different meanings of the word “fall”, demonstrating that ASL can sometimes convey meaning more clearly than spoken English.

#### Lissi et al. (2009)

The study examined the activities and strategies used by three deaf teachers to teach written language to Chilean deaf students in preschool, 1st, and 2nd grades using CHSL. It focused on the strategies’ effects on learning written Spanish, especially vocabulary and new words. Analysis of eight classroom video recordings (400 minutes total) showed that fingerspelling, pointing, and signs were frequently used to help students memorize and recall words and support writing. However, these micro-strategies were inconsistent, leading to the conclusion that a more systematic application of such strategies, including fingerspelling, would better aid students in learning written Spanish.

#### MacGlaughlin (2018)

MacGlaughlin's (2018) dissertation focuses on three college-educated deaf families who are bilingual in ASL and English, communicating with children aged 5 to 6 years old enrolled in preschool and kindergarten in the USA. The study investigated how deaf parents use fingerspelling with their children during communication, story-book reading, and free-writing activities. Using a grounded theory framework, the author collected data through interviews, background questionnaires, observations, and reflection journals from both the parents and their children. The study found that fingerspelling plays a crucial role in early language acquisition and serves as a foundational element for pre-literacy development. The families frequently employed fingerspelling and chaining, particularly during reading activities, which helped the deaf children develop reading behaviors and transition to print literacy. The author characterized fingerspelling as a distinctly deaf-centric epistemology and an essential resource for reading.

#### Roos (2004 *,*  2013*,*  2014)

Roos’ studies are based on the same dataset from her doctoral dissertation (Roos, 2004), which offers a more extensive exploration than her subsequent articles (Roos, 2013; Roos, 2014). The research is built on a longitudinal ethnographic study involving six deaf children aged 3:1 to 6:9 years, encompassing 48 hours of video recordings and fieldnotes from everyday interactions in preschool settings. The primary aim is to describe early literacy events among deaf children during their preschool years, focusing on their interactions and on meaning-making related to written language. The findings reveal that fingerspelling plays multiple roles, serving as a vital tool for reading and enhancing word knowledge and PA. In Roos (2013), six themes related to fingerspelling and literacy were identified, including the initial steps involved in learning fingerspelling and their connection to written words. Roos (2014) examined the daily use of fingerspelling from a sociocultural perspective, categorizing children’s fingerspelling into stages and demonstrating its role in play and identity. Collectively, Roos emphasizes the significance of fingerspelling in young deaf children’s interactions and literacy development.

#### Wang and Andrews (2021)

This study examines classroom activities during two literacy lessons involving DHH students in China. It begins with an overview of Pinyin’s history and its application as a pedagogical tool for teaching literacy to both hearing and deaf children. The research analyzes how teachers effectively utilize Pinyin in an oral education setting, detailing observations from video recordings of literacy instruction in grade 1 (n = 6 students) and grade 2 (n = 5 students). The findings reveal that Pinyin and fingerspelling (via the Pinyin manual alphabet) are frequently used alongside speech, airwriting, and sign, akin to chaining. The study highlights the phonological connection between Pinyin and fingerspelling, with Pinyin providing a visual representation of sounds for teachers. However, it emphasizes that DHH children lack prior knowledge of Chinese, using Pinyin and fingerspelling primarily as tools rather than as sources of PA. The authors call for further research into the effectiveness of fingerspelling in literacy instruction.

### Mixed methods studies

Four studies were identified as using mixed methods and are accounted for below: Miller et al. (2021)*,*  Padden and Ramsey (1998, 2000); and Scott, Hansen, and Lederberg et al. (2019).

#### Miller et al. (2021)

The study by Miller et al. (2021) investigated whether fingerspelling aids deaf children aged 4.2 to 6.0 years in developing orthographic knowledge despite limited phonological skills. The authors aimed to determine whether fingerspelling could help children form mental images of written words that connect to their meanings without relying on sound. Using a multi-probe single-subject design, the researchers tracked each child’s progress individually as they engaged in activities linking fingerspelling, written words, and meanings. Results indicated that three of the four children rapidly improved in recognizing and understanding both written and fingerspelled words. Additionally, they retained these skills in follow-up tests. The findings suggest that fingerspelling effectively establishes a connection between letters and meanings, acting like phonics for deaf children. Miller et al. advocate for early literacy interventions, starting as young as age four, to foster strong orthographic knowledge in prelingually deaf children and to emphasize the importance of active learning in developing literacy programs.

#### Padden and Ramsey (1998 *,*  2000)

Padden and Ramsey's (2000) book chapter revisits three studies initially reported in 1998, exploring the relationship between ASL and reading skills among deaf American children in elementary schools. The first study examined demographic factors, revealing that reading achievement was positively correlated with having deaf parents, early detection of deafness, and longer school attendance, while negatively correlating with additional needs. The second study assessed the impact of ASL skills on reading development in 31 deaf children across various grades, finding a correlation between ASL skills, fingerspelling proficiency, and reading abilities. This suggests that proficient readers utilize a combination of ASL and fingerspelling skills, although the direction of influence remains unclear. The third qualitative study analyzed reading instruction methods through longitudinal video recordings, highlighting varied reading behaviors such as the use of signs, fingerspelling, and mouthing words. Deaf teachers employed fingerspelling and chaining more than their hearing counterparts, reinforcing the importance of these techniques in successful reading instruction, yet the exact nature of their influence remains ambiguous.

#### Scott et al. (2019)

Scott et al. (2019) examine the relationship between fingerspelling and reading learning through a single-case research design in an instructional setting. The study involved six students divided into two groups—a kindergarten group (ages 6–7) and a first-grade group (ages 8–10)—all of whom used cochlear implants (CI) or hearing aids (HA). Three instructional conditions were tested: (a) both receptive and productive fingerspelling; (b) receptive fingerspelling only (via chaining); and (c) no fingerspelling, focusing solely on sign-print mapping.

The results did not clearly support the hypothesis that fingerspelling enhances word learning compared to other methods. However, post-test results indicated that fingerspelling might aid word retention. The authors noted limitations, including the diverse backgrounds of the participants, and suggested that future studies should include deaf students without delays. Additionally, the potential carryover of knowledge between conditions may have affected the outcomes. Overall, the authors emphasize the importance of explicit word learning instruction, aligning with the Dual Route Model of reading, which suggests both direct and indirect pathways to reading through fingerspelling, chaining, print, and signs.

### Summary of the reviewed literature

The 13 quantitative studies used direct assessments (reading skills, fingerspelling skills), indirect assessments (memory skills, phonological skills, and sign language skills), and teacher reports. The number of participants varied widely, from 16 to 336. The nine qualitative studies used observations, reports, interviews, and meta-discussions as their data sources; these studies focused on teachers’ perspectives and children’s fingerspelling practices. Participants ranged from 2 to 10, and they were generally younger (kindergarten or primary school students) than those in quantitative studies. Four studies used mixed methods, and two employed a traditional combination of quantitative and qualitative measures. In contrast, two others used “hybrid” experimental single-case designs for in-depth analyses of a few participants.

In addressing this study’s Research Question 1 (RQ1), “What is the relationship between fingerspelling and reading?”, most studies—specifically 12 out of 26, particularly the quantitative ones—indicated a positive correlation between fingerspelling and reading (Alvarado et al., 2007; Crume et al., 2021; Hanson et al., 1984; Haptonstall-Nykaza & Schick, 2007; Hile, 2010; Lederberg et al., 2019; Ormel et al., 2022; Padden & Ramsey, 1998, 2000; Puente et al., 2006; van Staden & le Roux, 2010). However, Harris and Beech (1998) did not find such a relationship. In some studies, fingerspelling and sign language knowledge may be intertwined as factors influencing reading (e.g., Alvarado et al., 2007; Hile, 2010; Padden & Ramsey, 1998, 2000; Puente et al., 2006). Several studies responding to RQ1 also addressed Research Question 2 (RQ2), “In what ways does fingerspelling promote reading skills?”, with 16 out of 26 studies participating. These studies suggest that fingerspelling promotes sublexical skills such as letter/grapheme knowledge and metalinguistic knowledge (Allen, 2015; Alvarado et al., 2007; Crume et al., 2021), acts as a visual bridge between sign and written language in various ways (Alvarado et al., 2007; Haptonstall-Nykaza & Schick, 2007; Hirsh-Pasek, 1986, 1987; Miller et al., 2021; Ormel et al., 2022; van Staden & le Roux, 2010), and supports language comprehension as well as word recognition and knowledge (Harris & Beech, 1998; Miller et al., 2021; Ormel et al., 2022; Puente et al., 2006; Scott et al., 2019). Additionally, several studies highlight fingerspelling as a crucial tool for facilitating orthographic skills and decoding (Hirsh-Pasek, 1986, 1987; Miller et al., 2021; Ormel et al., 2022; Padden & Ramsey, 1998, 2000). Some studies adopt a more holistic approach, viewing fingerspelling as part of the social interaction critical for literacy development (MacGlaughlin, 2018; Roos, 2004, 2013, 2014).

Research Question 3 (RQ3), “What is known about the use of fingerspelling in teaching reading?”, was addressed by 14 out of 26 studies, primarily qualitative ones. Several studies emphasize the need for training or explicit learning that includes fingerspelling to enhance children’s reading development (Hile, 2010; Hirsh-Pasek, 1986, 1987; Miller et al., 2021; Puente et al., 2006; Scott et al., 2019; van Staden & le Roux, 2010). Some studies describe the use of fingerspelling in classrooms or at home. For instance, Padden and Ramsey (1998, 2000) reported a higher use of fingerspelling among deaf teachers compared to hearing teachers in instruction. Other studies describe fingerspelling as a tool for chaining, sandwiching, or mediating between sign language and written language (Bailes, 1999; Crume, 2013; Erting & Pfau, 1997; Lissi et al., 2009; Roos, 2004, 2013, 2014; Wang & Andrews, 2021), with some advocating for more systematic use of fingerspelling in classrooms (e.g., Lissi et al., 2009). Additionally, some studies treat fingerspelling as part of a social construct for learning, play, and pre-literacy activities (MacGlaughlin, 2018; Roos, 2004, 2013, 2014), viewing it as an effective component of bilingual identity and learning (Crume, 2013) and a deaf-centric way of learning (MacGlaughlin, 2018; cf. Lee & Secora, 2022).

## Discussion

The studies covered in this scoping review originate from diverse time periods and theoretical frameworks, encompassing participants with varied linguistic backgrounds. Acknowledging the globally heterogeneous landscape of deaf education, as previously discussed, is paramount. Across the years, a range of methodologies and pedagogical orientations have shaped educational practices. Consequently, when considering the synthesis of these studies, it is essential to recognize that disparate outcomes may be attributable to variations in the languages to which children had access and in which they received instruction. For instance, studies failing to demonstrate a relationship between fingerspelling and reading may have included participants with impoverished linguistic foundations, potentially language-deprived children. Fingerspelling does not occur in isolation but is an integral component of sign language; thus, strong sign language proficiency is associated with enhanced fingerspelling skills. The inclusion of students with robust language skills may amplify observed positive correlations between fingerspelling and reading, contrasting findings derived from samples with weaker linguistic backgrounds. Research isolating fingerspelling as a discrete skill, rather than considering it within the broader context of sign language competence, may also yield diminished or attenuated associations.

Furthermore, while some studies explicitly assess sign languages or spoken languages, others report assessment of “language” without clear specification. Given the historical prevalence and enduring influence of oralist approaches, it is reasonable to infer that, in such instances, the assessment likely pertained to spoken language.

It should be said that fingerspelling was highlighted early on as a potential resource for supporting deaf students’ reading. As early as the 1950s, there was significant interest in using fingerspelling as an instructional method to teach reading to deaf students in the USA, most notably through the Rochester method. Although this approach was later abandoned (Morere & Roberts, 2012), recent research has highlighted the role of fingerspelling in promoting bilingualism in educational and family settings. Fingerspelling now plays an integral role in the chaining method and is a key component of the translanguaging sign framework (cf. Lee & Secora, 2022; Wang & Andrews, 2021, p. 459).

The reviewed studies reveal several key themes and findings that highlight the significance of fingerspelling in educational contexts. First, fingerspelling is consistently recognized as an effective bridge between sign language (ASL or other signed languages) and written language (e.g., Bailes, 1999; Crume, 2013; Erting & Pfau, 1997; Haptonstall-Nykaza & Schick, 2007; Lederberg et al., 2019; Miller et al., 2021). Many studies, including those by Miller et al. (2021) and Padden and Ramsey (1998, 2000), show that fingerspelling aids children in connecting letters with meanings, facilitating a better comprehension of written words. This is particularly beneficial for deaf children, as it helps establish a foundational understanding of literacy that might not rely solely on phonological skills.

It is important to remember that most reviewed studies (15 of 26) have focused on ASL and written English. It is not certain that the outcomes will be the same worldwide. For example, Puente et al. (2006) pointed out an interesting question related to phoneme–grapheme correspondence. Compared to English, Spanish has a higher phoneme–grapheme correspondence; i.e., it is more transparent compared to English, and the authors hypothesize that fingerspelling might be a stronger bridge to reading. Exploring different languages’ phoneme–grapheme correspondences and their “consequences” for fingerspelling as a bridge to reading merits further investigation.

Several of the reviewed studies show that fingerspelling plays a crucial role in enhancing orthographic knowledge (e.g., Crume et al., 2021; Haptonstall-Nykaza & Schick, 2007; Hile, 2010; Miller et al., 2021). Several studies also indicate that fingerspelling allows deaf children to improve their recognition and recall of written words, which can be particularly vital for those who may struggle with conventional phonological processing. For instance, Miller et al. (2021) demonstrated that fingerspelling assists in forming mental images of written words, thereby reinforcing their meanings without depending on sound.

The importance of fingerspelling also extends into vocabulary acquisition. Researchers often point out its significant role in understanding both English and other languages by promoting connections between signed and written forms (Erting & Pfau, 1997; Haptonstall-Nykaza & Schick, 2007; Lissi et al., 2009; MacGlaughlin, 2018; Miller et al., 2021). This reinforces the notion that fingerspelling is not just an isolated skill but a vital component in the broader context of language learning.

Additionally, the influence of sign language proficiency on reading skills is frequently noted (e.g., Alvarado et al., 2007; Crume et al., 2021; Lederberg et al., 2019; Padden & Ramsey, 1998, 2000). For example, Padden and Ramsey (2000) found that higher levels of ASL skills positively correlate with reading achievement. This suggests that fluency in ASL enhances fingerspelling abilities, which in turn supports overall literacy development. Effective instructional strategies that incorporate fingerspelling are a critical theme. Studies highlight techniques such as “chaining” (Bailes, 1999; Crume, 2013; Haptonstall-Nykaza & Schick, 2007; Scott et al., 2019) and “lexicalized fingerspelling” (Crume, 2013; Erting & Pfau, 1997; Haptonstall-Nykaza & Schick, 2007; Lederberg et al., 2019; MacGlaughlin, 2018) as methods that make learning more engaging and accessible for students.

However, while fingerspelling is widely recognized for its benefits according to the reviewed studies, some studies report mixed results regarding its effectiveness compared to other instructional methods. For example, Scott et al. (2019) noted that while fingerspelling might aid in word retention, it did not consistently show superiority over other instructional approaches. This highlights the need for more comprehensive research to fully understand the varied impacts of fingerspelling in literacy instruction.

Several studies have indicated that significant research is still needed in the field of fingerspelling and its relationship to reading development. For instance, Scott et al. (2019) emphasized the importance of conducting more research on reading interventions which incorporate fingerspelling to enhance literacy skills in deaf and hard-of-hearing children. Current evidence regarding effective interventions supporting deaf children’s literacy learning is limited. While the literature presents compelling evidence of a strong relationship between fingerspelling and reading skills, some studies specifically highlight fingerspelling skills—rather than sign language skills—as crucial for reading development (e.g., Lederberg et al., 2019; Stone et al., 2015). However, further research is needed to explore how these findings can be applied to teaching practices. One example in this area is the “Fingerspelling Our Way to Reading” program (Schick et al., 2018; Scott et al., 2019).

Regarding methodology, our review identified a notable lack of neurolinguistic data on children, in contrast to the data available for adults. Future research could address this gap by incorporating longitudinal studies that examine the development of fingerspelling and reading skills among deaf students. Previous studies, such as those by Padden and Ramsey (2000), often relied on correlation analyses, leaving the distinct relationships between fingerspelling, sign language, and reading skills unclear. For instance, it remains uncertain whether strong fingerspelling skills depend on proficiency in sign language or if they are influenced by reading skills (see, e.g., Padden & Ramsey, 2000, pp. 186–187). Utilizing a multivariate analysis or linear regression model could help clarify these relationships, particularly for children who are in the process of learning to read. While Stone et al. (2015) address some of these questions in adults, it would be intriguing to investigate whether similar findings apply to deaf children learning to read. Additionally, this raises the question of whether there is a strictly mutual relationship between fingerspelling and reading skills.

Previous research has included deaf participants from diverse age groups and backgrounds, primarily focusing on deaf signing students. However, advancements in hearing technology and the increased use of cochlear implants have allowed more students to experience hearing, as observed in countries like Sweden. This shift presents an opportunity to explore how fingerspelling interacts with hearing and the process of learning to read. Studies on bilingual deaf children with cochlear implants have revealed specific patterns similar to those seen in the children of deaf adults, particularly concerning spelling in writing (Gärdenfors et al., 2019) and spoken language acquisition (Davidson et al., 2014). These findings create promising avenues for future research to investigate the relationships between fingerspelling, sign language, and spoken/written language in relation to reading development among deaf students.

Taken together, the common themes identified in our review underscore the crucial role that fingerspelling plays in enhancing literacy and language development among deaf children. The integration of fingerspelling within educational practices supports a holistic approach that combines various modes of communication and instructional strategies, as reflected by the breadth of the research in this area.

## Footnotes

1 By using “phonological awareness” in this paper, we refer to such awareness generally, although our study primarily focuses on sound-based phonological awareness. Sign-based phonological awareness may also be relevant, and our scoping review includes studies on fingerspelling regardless of whether they are based on spoken or sign language–based phonological awareness.
